# Impact of chronic obstructive pulmonary disease on mortality in elderly patients with hip fracture: A retrospective cohort study

**DOI:** 10.1371/journal.pone.0340474

**Published:** 2026-01-06

**Authors:** Jiaxuan Zhu, Yu Chang, Xiaomin Wang, Yuying Li, Fan Yang, Yuwei Shi, Xiuguo Zhang

**Affiliations:** Department of Nursing, The Third Hospital of Hebei Medical University, Shijiazhuang, People’s Republic of China; University of Health Sciences, Beyhekim Training and Research Hospital, TÜRKIYE

## Abstract

**Objective:**

Chronic obstructive pulmonary disease (COPD) is thought to increase mortality risk in elderly patients with hip fracture, but its independent effect remains unclear. This study aimed to determine whether COPD is an independent predictor of mortality in this population.

**Methods:**

A retrospective cohort study was conducted on elderly patients with hip fractures. Through propensity score matching, confounding factors between patients with chronic obstructive pulmonary disease (COPD) and those without COPD were balanced. LASSO and Cox regression methods were used to identify predictors of mortality. The performance of the model was evaluated via ROC curves, calibration curves, and decision curve analysis. Restrictive cubic spline analysis and subgroup analysis were also performed to assess nonlinear effects and interactions.

**Results:**

A total of 151 (11.08%) of the 1362 participants who were enrolled in the trial had COPD. After PSM, 537 data points were analysed. The COPD group presented a significantly greater incidence of postoperative respiratory failure (6.04%) and delirium (12.08%) and significantly greater 1-year mortality (22.82% vs. 5.93%). COPD was identified as a strong independent predictor of mortality (HR 7.291, 95% CI: 3.619 ~ 14.691). Other independent risk factors included age (HR 1.066, 95% CI: 1.016 ~ 1.118), HCT (HR 0.922, 95% CI: 0.872 ~ 0.975), CAR (HR 1.476, 95% CI: 1.209 ~ 1.801), and BNP (HR 1.001, 95% CI: 1.000 ~ 1.001). The prediction model showed good predictive efficiency, achieving an AUC of 0.834 (95% CI: 0.766, 0.902) in the training set and 0.892 (95% CI: 0.819, 0.965) in the validation set. Restricted cubic splines confirmed linear relationships between continuous predictors and mortality risk (all P values for nonlinearity > 0.05). Subgroup analysis revealed a significant interaction effect between sex and COPD (p = 0.028).

**Conclusions:**

COPD is a strong independent risk factor for 1-year mortality in elderly hip fracture patients. The developed prediction model can help clinicians identify high-risk patients early and implement personalized management strategies.

## Introduction

As the population ages, there will be an estimated 2.6 million hip fractures by 2025, increasing to 4.5 million by 2050 [1]. The hospitalization of elderly patients suffering from hip fractures not only places considerable psychological pressure on individuals and their families but also generates a significant economic burden. Moreover, elderly individuals often have multiple coexisting chronic diseases, which increase their susceptibility to complications following hip fracture surgery, including infections, thrombosis, and heart and lung failure, thereby substantially increasing patient mortality rates [2].

Chronic obstructive pulmonary disease (COPD) is a heterogeneous pulmonary disease caused mainly by smoking and is characterized by chronic respiratory symptoms and airflow limitations that cannot be completely reversed [3]. Notably, while COPD is a primarily progressive lung disease, it presents with systemic manifestations, including osteoporosis [4]. The rate of hip fractures among COPD patients is 649 cases per 10,000 person-years [5]. In addition to an increased risk of surgery, a longer hospital stay, and financial hardship, elderly patients with hip fractures complicated by COPD also face a number of postoperative complications, including septic shock, venous thrombosis, respiratory failure, and postoperative pulmonary infection [6–8]. A study evaluating hip fracture data in the United Kingdom from 2010–2015 revealed that the 1-year mortality rate among patients with coexisting COPD was as high as 35.3% [9]. COPD patients suffer from impaired lung function and underlying chronic inflammation. This makes these patients more prone to postoperative complications, leading to extended hospital stays and increased risk of postoperative mortality [10]. According to Ozel et al., who monitored 275 elderly patients who had hip fractures, COPD was an independent predictor of death one year following surgery [11]. Therefore, their results may have certain biases. De Luise et al. reported that individuals with COPD have a 60–70% increased risk of mortality after hip fracture compared with those without COPD [12]. This study did not consider the influence of laboratory indicators on the results, which to some extent limits the comprehensiveness and accuracy of its results.

Consequently, this study aimed to achieve two principal objectives: 1) to rigorously assess the association between chronic obstructive pulmonary disease (COPD) and 1-year mortality in a comprehensively characterized cohort of elderly hip fracture patients, employing propensity score matching (PSM) to mitigate confounding biases; and 2) to develop and validate a personalized risk prediction model incorporating both clinical parameters and specific laboratory biomarkers, thereby enhancing the precision of high-risk patient identification.

## Materials and methods

### Study population

We collected data from the geriatric orthopedics department at Hebei Medical University Third Hospital’s Trauma Emergency Center, whose injuries occurred between January 2020 and December 2022. The data were accessed and analysed for research purposes in April 2024. Data were retrospectively collected from individuals aged 65 years and older who had undergone hip fracture surgery. Individuals with (1) multiple fractures, (2) pathological fractures, (3) ancient fractures, or (4) loss to follow-up were not included in the study. We obtained approval from the Ethics Review Committee of Hebei Medical University Third Hospital (2024-032-1) and adhered to the Helsinki Declaration. As this was a retrospective study, the requirement for informed consent was waived. To protect confidentiality, we analysed only deidentified data and had no access to any patient identifiers.

### Data collection

The indicators collected included basic demographic characteristics, laboratory indicators, surgery-related indicators and postoperative complications. Age, sex, BMI, fracture type, duration from accident to hospitalization, duration from hospitalization to surgery, and comorbidities were among the fundamental demographic factors. The laboratory indicators included hemoglobin (HB), electrolytes, white blood cell (WBC) count, red blood cell (RBC) count, hematocrit (HCT), brain natriuretic peptide (BNP), sodium (NA), potassium (K), the neutrophil-to-lymphocyte ratio (NLR), and C-reactive protein (CRP). Indicators pertaining to surgery included the type of operation, anaesthesia technique, ASA classification, length of surgery, and volume of intraoperative fluid infusion. Anaemia, pneumonia, pneumonia, urinary tract infection, respiratory failure, heart failure (HF), acute myocardial infarction (AMI), acute cerebral infarction (ACI), delirium, atrial fibrillation (AF), and deep venous thrombosis (DVT) are among the postoperative consequences. The above indicators were derived through a review of medical records. All-cause mortality at one year was the main outcome of interest. Complications following surgery were the secondary outcomes. Telephone follow-up was conducted approximately 1 year after surgery to assess patient survival.

### Definition of COPD

According to the diagnostic criteria of the Global Initiative for Chronic Obstructive Lung Disease (GOLD), the diagnosis of COPD is based on postbronchodilator spirometry (forced expiratory volume in 1 second to forced vital capacity ratio, FEV1/FVC < 0.70) [3]. This retrospective study is based on medical records, and COPD patients were identified through clear physician diagnosis records, namely, the diagnoses of “chronic obstructive pulmonary disease (COPD)”, “emphysema”, or “chronic bronchitis” recorded in the medical records.

### Statistical methods

The statistical analysis was conducted via IBM SPSS Statistics version 26.0 and R software version 4.4.0. For properly distributed continuous variables, the means ± standard deviations were used, whereas medians and quartiles were used for nonnormally distributed variables. For group comparisons, regularly distributed data were analysed via independent sample t tests, whereas nonnormally distributed data were evaluated via the Mann‒Whitney U test. Fisher’s exact test or the chi-square test was used to evaluate count data, which are presented as frequencies and percentages.

To balance baseline differences between the chronic obstructive pulmonary disease (COPD) and non-COPD groups, 1:3 nearest neighbor propensity score matching was performed with a calliper value of 0.2. The matching variables included sex, body mass index (BMI), time from injury to hospital admission, hypertension status, and surgical type. Survival curves were plotted via the Kaplan–Meier method, and survival differences between groups were compared via the log-rank test.

The matched samples were divided into a training set and a validation set at a ratio of 7:3. Predictors associated with survival were screened via least absolute shrinkage and selection operator (LASSO) regression, and those selected were incorporated into a multivariate Cox proportional hazards model to develop a predictive model. A nomogram was constructed for visualization. Collinearity among variables was examined via the variance inflation factor (VIF), with a VIF < 5 indicating no significant multicollinearity. The discriminative ability of the model was evaluated via the area under the receiver operating characteristic curve (AUC). Calibration curves were plotted to assess the consistency between the predicted and observed outcomes, and decision curve analysis was applied to evaluate clinical utility. Correlations among variables were visualized via a heatmap.

Restricted cubic splines (RCSs) were used to model the relationships between continuous variables and hazard ratios (HRs), with the number of knots determined by minimizing the Akaike information criterion (AIC). The threshold age with an HR of 1 was used to categorize patients into two groups: < 80 years and ≥80 years. The same method was applied to determine the optimal cut-off values for HCT, CAR, and BNP (see S1 Fig). Kaplan–Meier survival curves were generated on the basis of these cut-off values. Furthermore, subgroup analyses were conducted on the basis of age, sex, fracture type, comorbidities, surgical type, and ASA score via Cox regression. Interaction terms were introduced to examine whether the effect of COPD on prognosis differed significantly across subgroups. A two-sided P value < 0.05 was considered statistically significant.

## Results

### Baseline characteristics

**Fig 1:**
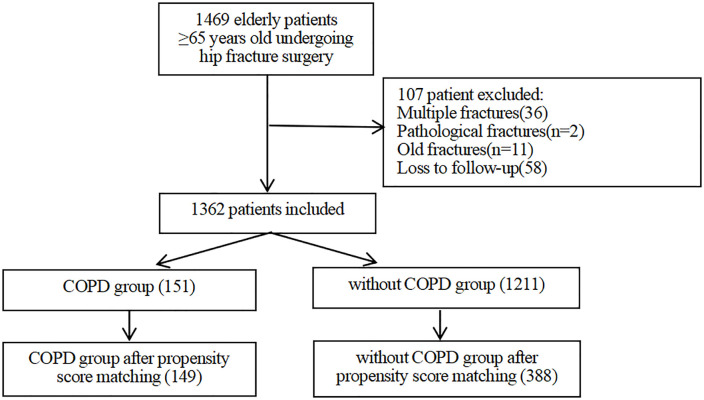
Flow chart of patient screening.

A total of 1469 senior individuals with hip fractures who were 65 years of age or older were included in our study. A total of 107 individuals were excluded because they did not meet the inclusion criteria. Among them, 58 patients were excluded because they were lost to follow-up through telephone contact. Ultimately, 1,362 patients remained in the study cohort (as shown in the inclusion and exclusion process flowchart in Fig 1). Table 1 summarizes the characteristics of the patients. Among the 1362 patients, 151 (11.08%) had a preexisting diagnosis of COPD. The patients had a median age of 80 years (74,85), and 71.59% were female. The sex distribution among these elderly hip fracture patients with COPD was predominantly male (62.91%). Patients in the COPD group had a lower BMI, a longer time from injury to admission, and a lower incidence of hypertension. Compared with those in the non-COPD group, the elderly hip fracture patients in the COPD group had fewer internal fixations and a greater NLR. After controlling for confounding variables via PSM, 149 and 388 patients were included in the COPD and without COPD groups, respectively. After propensity score matching, the standardized mean differences (SMDs) of all variables were ≤ 0.136 (far below the threshold of 0.2), and all P values were > 0.05 (Table 1).

**Table 1:** Baseline characteristics of elderly patients with hip fractures before and after PSM.

Variable	Before PSM					After PSM				
	Total (n = 1362)	Without COPD (n = 1211)	COPD (n = 151)	SMD	*P*	Total (n = 537)	Without COPD (n = 388)	COPD (n = 149)	SMD	*P*
Age, years	80.00 (74.00, 85.00)	80.00 (73.00, 85.00)	80.00 (75.00, 85.00)	0.065	0.474	80.00 (75.00, 85.00)	80.00 (74.00, 85.00)	80.00 (75.00, 85.00)	−0.013	0.904
Sex, n (%)					**<0.001**					0.350
Female	975 (71.59)	919 (75.89)	56 (37.09)	−0.803		219 (40.78)	163 (42.01)	56 (37.58)	−0.091	
Male	387 (28.41)	292 (24.11)	95 (62.91)	0.803		318 (59.22)	225 (57.99)	93 (62.42)	0.091	
BMI, kg/m^2^	23.52 (20.81, 26.12)	23.61 (21.23, 26.37)	22.22 (19.59, 24.49)	−0.349	**<0.001**	22.86 (20.20, 25.10)	22.89 (20.55, 25.39)	22.45 (19.59, 24.49)	−0.124	0.138
Type of fracture, n (%)					0.073					0.254
Femoral neck fracture	646 (47.43)	564 (46.57)	82 (54.30)	0.155		267 (49.72)	187 (48.20)	80 (53.69)	0.110	
Intertrochanteric fracture	716 (52.57)	647 (53.43)	69 (45.70)	−0.155		270 (50.28)	201 (51.80)	69 (46.31)	−0.110	
The time from injury to admission,h	24.00 (12.00, 49.50)	18.00 (12.00, 48.00)	24.00 (12.00, 84.00)	0.059	**0.016**	24.00 (12.00, 72.00)	24.00 (12.00, 72.00)	24.00 (12.00, 72.00)	0.045	0.154
The time from admission to surgery,h	96.00 (72.00, 144.00)	96.00 (72.00, 144.00)	108.00 (72.00, 156.00)	0.170	0.214	108.00 (72.00, 144.00)	108.00 (72.00, 144.00)	108.00 (72.00, 156.00)	0.073	0.705
Hypertension, n (%)					**0.005**					0.352
Yes	733 (53.82)	668 (55.16)	65 (43.05)	−0.245		248 (46.18)	184 (47.42)	64 (42.95)	−0.090	
No	629 (46.18)	543 (44.84)	86 (56.95)	0.245		289 (53.82)	204 (52.58)	85 (57.05)	0.090	
Coronary artery disease, n (%)					0.706					0.913
Yes	351 (25.77)	314 (25.93)	37 (24.50)	−0.033		128 (23.84)	92 (23.71)	36 (24.16)	0.011	
No	1011 (74.23)	897 (74.07)	114 (75.50)	0.033		409 (76.16)	296 (76.29)	113 (75.84)	−0.011	
Diabetes, n (%)					0.053					0.983
Yes	389 (28.56)	356 (29.40)	33 (21.85)	−0.183		115 (21.42)	83 (21.39)	32 (21.48)	0.002	
No	973 (71.44)	855 (70.60)	118 (78.15)	0.183		422 (78.58)	305 (78.61)	117 (78.52)	−0.002	
Cerebral infarction, n (%)					0.426					0.869
Yes	698 (51.25)	616 (50.87)	82 (54.30)	0.069		295 (54.93)	214 (55.15)	81 (54.36)	−0.016	
No	664 (48.75)	595 (49.13)	69 (45.70)	−0.069		242 (45.07)	174 (44.85)	68 (45.64)	0.016	
Pulmonary arterial hypertension, n (%)					0.265					0.780
Yes	73 (5.36)	62 (5.12)	11 (7.28)	0.083		37 (6.89)	26 (6.70)	11 (7.38)	0.026	
No	1289 (94.64)	1149 (94.88)	140 (92.72)	−0.083		500 (93.11)	362 (93.30)	138 (92.62)	−0.026	
Surgical type, n (%)					**0.014**					0.155
Joint replacement	525 (38.55)	453 (37.41)	72 (47.68)	0.206		226 (42.09)	156 (40.21)	70 (46.98)	0.136	
Internal fixation	837 (61.45)	758 (62.59)	79 (52.32)	−0.206		311 (57.91)	232 (59.79)	79 (53.02)	−0.136	
Method of anaesthesia, n (%)					0.189					0.704
Intraspinal anaesthesia	389 (28.56)	339 (27.99)	50 (33.11)	0.109		170 (31.66)	121 (31.19)	49 (32.89)	0.036	
General anaesthesia	973 (71.44)	872 (72.01)	101 (66.89)	−0.109		367 (68.34)	267 (68.81)	100 (67.11)	−0.036	
ASA, n (%)					0.056					0.483
Ⅰ-Ⅱ	740 (54.33)	669 (55.24)	71 (47.02)	−0.165		269 (50.09)	198 (51.03)	71 (47.65)	−0.068	
Ⅲ-Ⅳ	622 (45.67)	542 (44.76)	80 (52.98)	0.165		268 (49.91)	190 (48.97)	78 (52.35)	0.068	
Duration of surgery,h	90.00 (70.00, 115.00)	90.00 (70.00, 115.00)	90.00 (70.50, 115.50)	0.122	0.280	90.00 (75.00, 118.00)	90.00 (75.00, 118.50)	90.00 (71.00, 111.00)	0.048	0.699
Intraoperative fluid infusion volume, ml	1300.00 (1000.00, 1650.00)	1300.00 (1000.00, 1650.00)	1350.00 (1000.00, 1650.00)	0.094	0.256	1300.00 (1000.00, 1700.00)	1300.00 (1000.00, 1700.00)	1350.00 (1000.00, 1700.00)	0.006	0.862
HB, g/L	112.00 (100.48, 124.00)	112.00 (101.00, 124.00)	113.00 (96.85, 126.75)	0.029	0.882	113.30 (101.01, 126.00)	113.55 (102.00, 125.03)	112.80 (96.70, 126.90)	−0.037	0.575
WBC, 10^9^/L	6.89 (5.77, 8.73)	6.89 (5.80, 8.70)	6.93 (5.61, 9.18)	0.076	0.942	6.88 (5.68, 8.95)	6.84 (5.70, 8.92)	6.92 (5.60, 9.14)	0.039	0.982
RBC, 10^12^/L	3.97 (3.40, 6.55)	3.98 (3.42, 6.87)	3.86 (3.30, 5.61)	−0.079	0.169	3.99 (3.37, 5.96)	4.00 (3.43, 6.15)	3.86 (3.30, 5.65)	−0.052	0.300
HCT, %	33.40 (29.80, 36.80)	33.40 (29.88, 36.77)	33.42 (28.72, 37.00)	0.030	0.926	33.61 (29.70, 37.30)	33.71 (30.10, 37.30)	33.40 (28.65, 37.04)	−0.023	0.595
Sodium, mmol/L	138.20 (135.90, 140.04)	138.30 (135.96, 140.09)	137.89 (134.69, 139.79)	−0.118	0.125	137.80 (135.26, 139.70)	137.77 (135.48, 139.70)	137.89 (134.57, 139.78)	−0.050	0.915
Potassium, mmol/L	3.85 (3.60, 4.10)	3.85 (3.60, 4.10)	3.86 (3.59, 4.16)	0.099	0.463	3.88 (3.62, 4.12)	3.88 (3.65, 4.11)	3.86 (3.58, 4.16)	−0.016	0.763
BNP, pg/mL	144.00 (73.00, 265.00)	142.00 (70.00, 261.00)	153.00 (87.50, 286.00)	0.177	0.052	154.00 (84.00, 279.00)	155.50 (81.00, 277.50)	153.00 (87.00, 282.00)	0.050	0.596
NLR	5.20 (3.57, 7.40)	5.09 (3.50, 7.37)	6.19 (4.32, 8.77)	0.274	**<.001**	5.97 (3.99, 8.49)	5.94 (3.94, 8.48)	6.08 (4.30, 8.50)	0.036	0.640
CAR	1.31 (0.72, 1.97)	1.32 (0.71, 1.96)	1.28 (0.79, 2.17)	0.017	0.706	1.38 (0.76, 2.15)	1.44 (0.75, 2.13)	1.28 (0.79, 2.17)	−0.105	0.479

Values are presented as the mean±standard deviation, median and quartiles, or number (percentage) as appropriate, SD Standard deviation.

Abbreviation: ASA:American Society of Anesthesiologists Classification; HB: hemoglobin; WBC: white blood cell; RBC: red blood cell; HCT: hematocrit; BNP: Brain natriuretic peptide; NLR:Neutrophil/lymphocyte count ratio; CAR: C-reactive protein/albumin ratio.

### Postoperative complications and 1-year all-cause mortality after PSM

**Fig 2:**
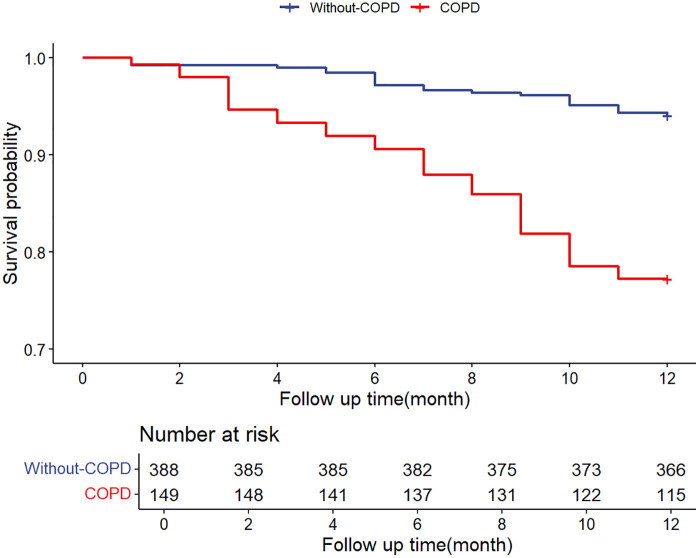
The Kaplan–Meier curve comparing 1-year all-cause mortality between patients with and without COPD.

Table 2 presents the outcomes of postoperative complications and death rates for aged patients with hip fractures. Elderly individuals with hip fractures who also had COPD presented a statistically significant (P < 0.05) increased risk of postoperative respiratory failure (6.04% vs 1.55%, P = 0.011) and delirium (12.08% vs 4.38%, P = 0.001). The incidence of other complications after surgery did not significantly differ between the two groups. We also found that anaemia was the most prevalent complication in both groups. Upon completion of the follow-up period, the death rate was greater in the COPD group than in the non-COPD group (22.82% vs 5.93%, P < 0.001). This was illustrated through the Kaplan‒Meier survival curve depicted in Fig 2 (log rank p < 0.001, hazard ratio (HR) 95% CI: 4.213, 2.481 ~ 7.153).

**Table 2:** Comparison of postoperative complications between the two groups after PSM.

Variables	Total (n = 537)	Without COPD (n = 388)	COPD (n = 149)	*P*
Pneumonia, n(%)				0.406
Yes	110 (20.48)	76 (19.59)	34 (22.82)	
No	427 (79.52)	312 (80.41)	115 (77.18)	
Anemia, n(%)				0.612
Yes	207 (38.55)	147 (37.89)	60 (40.27)	
No	330 (61.45)	241 (62.11)	89 (59.73)	
Urinary system infection, n(%)				0.662
Yes	39 (7.26)	27 (6.96)	12 (8.05)	
No	498 (92.74)	361 (93.04)	137 (91.95)	
Respiratory failure, n(%)				**0.011**
Yes	15 (2.79)	6 (1.55)	9 (6.04)	
No	522 (97.21)	382 (98.45)	140 (93.96)	
Heart failure, n(%)				0.633
Yes	147 (27.37)	104 (26.80)	43 (28.86)	
No	390 (72.63)	284 (73.20)	106 (71.14)	
Acute cerebral infarction, n(%)				0.123
Yes	86 (16.01)	68 (17.53)	18 (12.08)	
No	451 (83.99)	320 (82.47)	131 (87.92)	
Acute myocardial infarction, n(%)				0.235
Yes	13 (2.42)	7 (1.80)	6 (4.03)	
No	524 (97.58)	381 (98.20)	143 (95.97)	
Traumatic cholecystitis, n(%)				0.998
Yes	9 (1.68)	6 (1.55)	3 (2.01)	
No	528 (98.32)	382 (98.45)	146 (97.99)	
Delirium, n(%)				**0.001**
Yes	35 (6.52)	17 (4.38)	18 (12.08)	
No	502 (93.48)	371 (95.62)	131 (87.92)	
Atrial fibrillation, n(%)				0.287
Yes	40 (7.45)	26 (6.70)	14 (9.40)	
No	497 (92.55)	362 (93.30)	135 (90.60)	
DVT, n(%)				0.076
Yes	147 (27.37)	98 (25.26)	49 (32.89)	
No	390 (72.63)	290 (74.74)	100 (67.11)	
1-year all-cause mortality,n(%)				**<0.001**
Yes	57 (10.61)	23 (5.93)	34 (22.82)	
No	480(89.39)	365 (94.07)	115 (77.18)	

Values are presented as number (percentage).

DVT: deep venous thrombosis.

### Risk variables and evaluation of a nomogram model’s predictive accuracy for 1-year death in older patients with hip fractures

A total of 537 participants were randomly split into training and validation sets at a 7:3 ratio. The prevalence rates of 1-year death were 10.90% (41/376) and 9.94% (16/161) in the training and validation sets, respectively. The two groups had comparable baseline characteristics. The participant characteristics are detailed in S1 Table.

**Fig 3:**
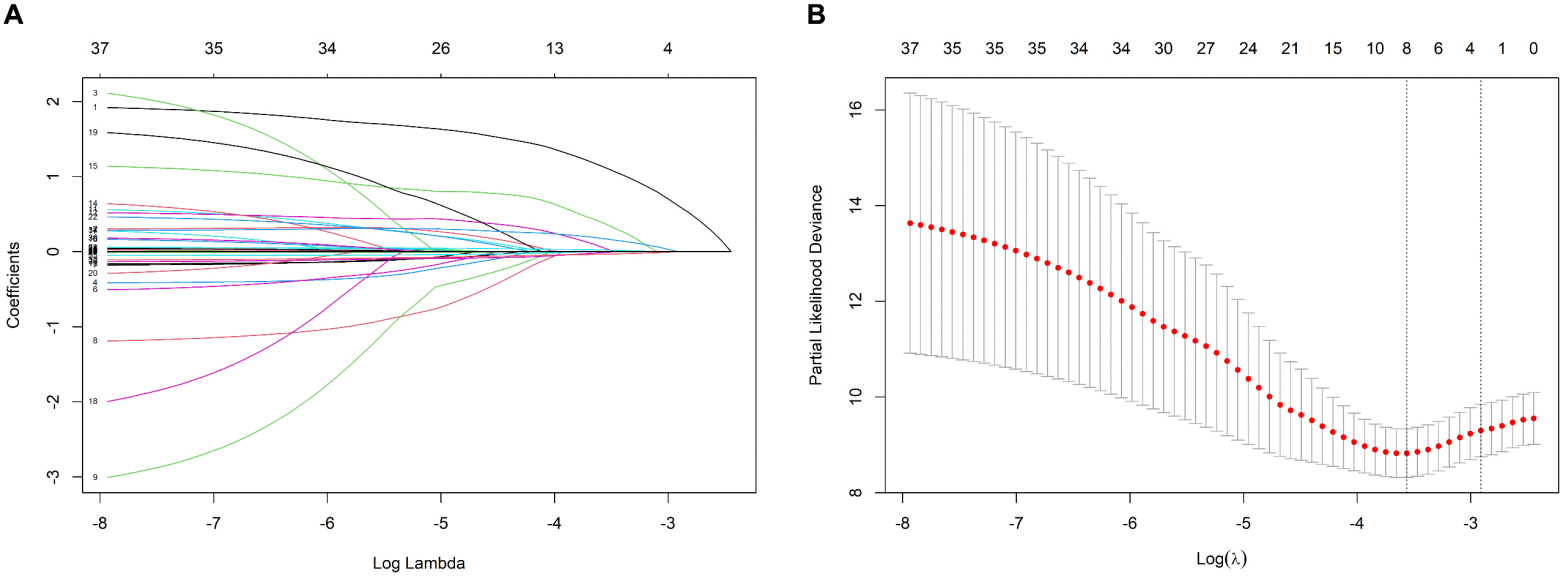
Data statistics and clinical feature selection via LASSO regression. **(A)** The 37 features of the LASSO coefficient curve. A coefficient profile was generated from the log(lambda) sequence. **(B)** Results of the cross-validation procedure. The left vertical line signifies λ min, whereas the right vertical line denotes λ 1 se. λ min is the λ value associated with the lowest mean squared error (MSE) across all the λ values. Conversely, λ 1 se represents the λ value chosen for the simplest and best model, identified through cross-validation within a specified range of λ min’s squared difference.

**Fig 4:**
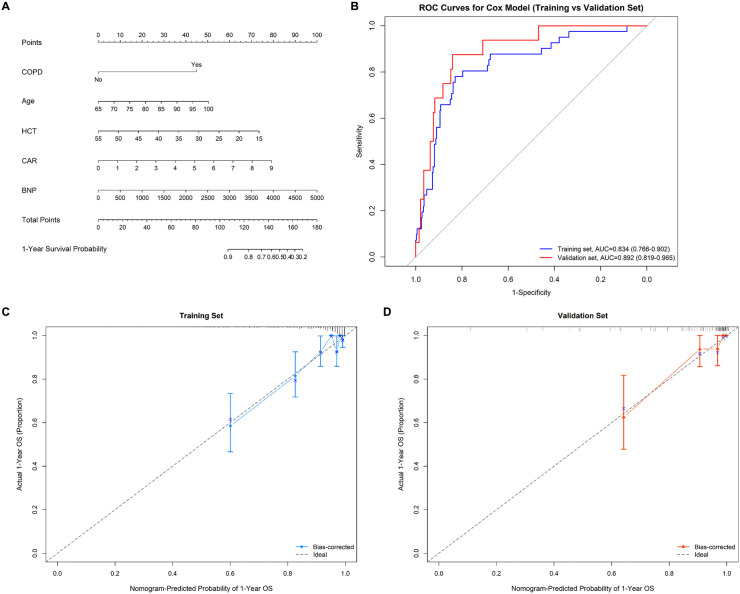
Predictive performance of the nomogram for 1-year all-cause mortality in elderly hip fracture patients after PSM. **(A)** Nomogram representation for predicting 1-year all-cause mortality in elderly patients with hip fracture after PSM. HCT: hematocrit; CAR: C-reactive protein/albumin ratio; BNP: brain natriuretic peptide; **(B)** ROC curves of the training set and the validation set. **(C)** Calibration curve of the training set. **(D)** Calibration curve of the validation set.

**Fig 5:**
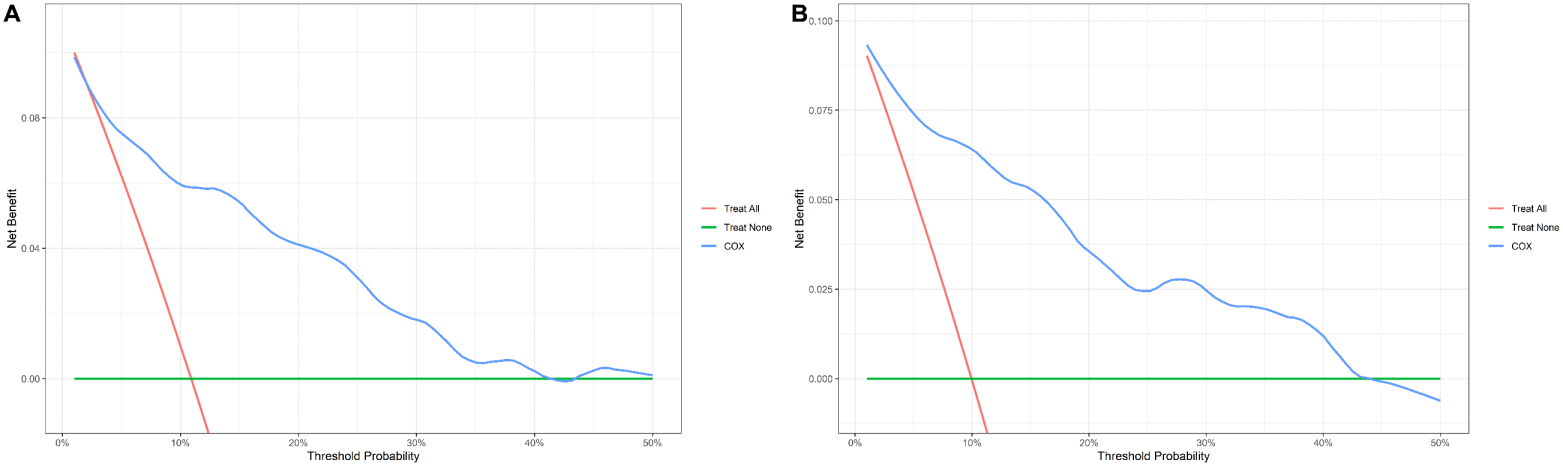
Decision curve analysis (DCA) of the model. (A) DCA curve of the training set; (B) DCA curve of the validation set.

**Fig 6:**
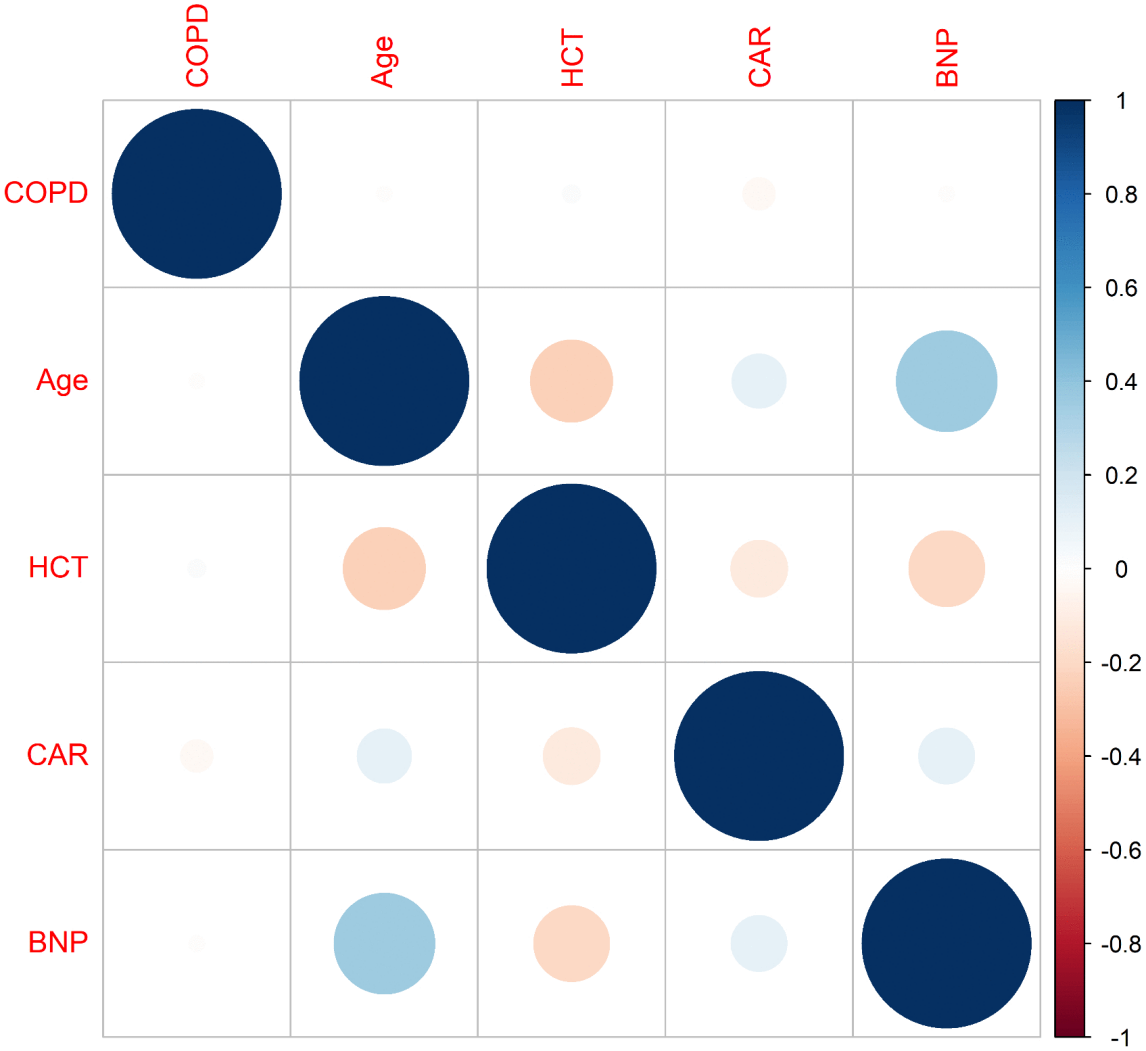
Correlation heatmap of the parameters. Blue indicates a positive correlation, and red indicates a negative correlation.

Using LASSO regression (λ min criterion), 8 variables were selected for further analysis (Fig 3). Multivariate Cox analysis revealed the following independent predictors of 1-year all-cause mortality in elderly hip fracture patients after PSM: age (HR 1.066, 95% CI: 1.016 ~ 1.118), HCT (HR 0.922, 95% CI: 0.872 ~ 0.975), CAR (HR 1.476, 95% CI: 1.209 ~ 1.801), and BNP (HR 1.001, 95% CI: 1.000 ~ 1.001) (Table 3). All the variables had VIF values well below 5, indicating no significant multicollinearity. A restricted cubic spline function was used to construct a nonlinear relationship between these predictors and mortality. The nonlinear test results revealed that the P values were all greater than 0.05 (age 0.213, BNP 0.086, CAR 0.343, and HCT 0.251), indicating a linear association between each variable and the risk of death (S1A–S1D Fig). On the basis of these analyses, the following optimal critical values were obtained: age (80 years), HCT (33.61%), CAR (1.38), and BNP (154 pg/mL). The Kaplan–Meier curves stratified by these cut-offs are shown in S1E–S1H Fig. We created a graphic nomogram (Fig 4A) to show how each condition affects the outcomes of elderly people who have hip fractures. The prediction model showed good predictive efficiency, achieving an AUC of 0.834 (95% CI: 0.766, 0.902) in the training set and 0.892 (95% CI: 0.819, 0.965) in the validation set (Fig 4B). The calibration curves of the training set and the validation set indicate that this model performs well (Fig 4C, 4D). Decision curve analysis confirmed its clinical utility within threshold probabilities of 5–40% (Fig 5). Correlation analysis revealed that age exhibited a mild positive correlation with BNP levels (brain natriuretic peptide) and a weak positive correlation with the CAR (C-reactive protein-to-albumin ratio). Furthermore, a weak positive correlation was identified between CAR and BNP. In contrast, age demonstrated a mild negative correlation with HCT (hematocrit), whereas HCT showed mild negative correlations with both CAR and BNP (Fig 6).

**Table 3:** Factors predicting 1-year all-cause mortality in elderly patients with hip fractures.

Variable	β	S.E	Z	P	HR	95% CI
COPD	1.987	0.357	5.559	<0.001	7.291	3.619 ~ 14.691
Age	0.064	0.024	2.645	0.008	1.066	1.016 ~ 1.118
HCT	−0.081	0.028	2.863	0.004	0.922	0.872 ~ 0.975
CAR	0.389	0.102	3.825	<0.001	1.476	1.209 ~ 1.801
BNP	0.001	0.000	3.789	<0.001	1.001	1.000 ~ 1.001

HR: Hazards Ratio, CI: Confidence Interval; COPD: Chronic obstructive pulmonary disease; HCT: hematocrit; CAR: C-reactive protein/albumin ratio; BNP: Brain natriuretic peptide.

### Subgroup analyses

**Fig 7:**
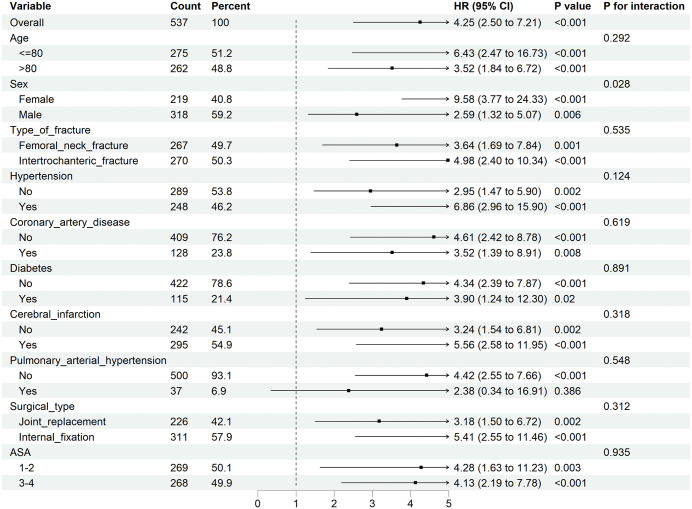
Subgroup analyses of the associations between COPD incidence and 1-year all-cause mortality in patients who underwent hip fracture surgery.

As shown in Fig 7, there was an interaction effect between sex (p = 0.028) and COPD in different subgroups of elderly patients with hip fracture. Both the male (p = 0.006) and female (p < 0.001) subgroups were significantly associated with COPD and 1-year mortality. The positive association between COPD and one-year mortality was more pronounced in elderly female patients with hip fracture. No significant interactions were found between COPD and other subgroup factors. These results suggest that COPD is an independent risk factor for increased mortality in elderly hip fracture patients.

## Discussion

This study identified chronic obstructive pulmonary disease (COPD) as a powerful independent risk factor for one-year mortality in elderly patients following hip fracture surgery, with an adjusted hazard ratio of 7.291. Patients with COPD also experienced significantly higher rates of postoperative respiratory failure and delirium. Furthermore, advanced age, an elevated C-reactive protein-to-albumin ratio (CAR), and elevated B-type natriuretic peptide (BNP) at admission were independent predictors of increased mortality, whereas a higher hematocrit (HCT) level was protective. The predictive model constructed on the basis of these risk factors provides a practical tool for clinical risk stratification.

Our analysis confirmed that COPD is a powerful independent predictor of one-year mortality in elderly hip fracture patients (adjusted HR 7.291, 95% CI: 3.619–14.691). This significantly elevated risk is likely mediated through a greater burden of postoperative complications, particularly respiratory failure and delirium, which themselves are strong drivers of poor outcomes. The increased incidence of these complications in COPD patients may stem from the interplay of long-term systemic inflammation, impaired gas exchange, and reduced physiological reserve, which collectively increase vulnerability to surgical stress [13,14].

The pathophysiological mechanisms linking COPD to increased mortality involve several synergistic pathways that explain both the risk of complications and direct systemic deterioration. First, COPD is characterized by persistent chronic inflammation of the airways, lung parenchyma, and pulmonary vasculature [15–17]. In addition to driving local lung damage, these inflammatory mediators exacerbate the systemic inflammatory response postfracture, increasing the risk of cardiovascular events and multiorgan dysfunction [18]. Second, inflammatory processes disrupt the protease–antiprotease balance, leading to parenchymal tissue destruction and emphysematous changes, which further compromise lung function and reserve [19,20]. Third, marked oxidative stress in COPD causes direct cellular damage and accelerates pulmonary decline while also potentiating systemic inflammatory responses [21,22]. Together, these mechanisms lead to the underlying frailty state in elderly patients with hip fractures and COPD. This compromised state not only predisposes patients to postoperative respiratory failure due to diminished pulmonary reserve and vulnerability to atelectasis or pneumonia [13,23] but also contributes to delirium through chronic hypoxemia, CO₂ retention, and neuroinflammation [24–26]. Thus, the elevated one-year mortality observed in COPD patients is the culmination of this preexisting pathological burden, which is acutely exacerbated by surgical stress, leading to a more complicated postoperative course and impaired recovery [27,28].

Older age (HR 1.066, 95% CI: 1.016 ~ 1.118) increases the chance of death following hip fracture surgery in elderly patients [29,30]. According to Ogawa et al., older patients with hip fractures have higher rates of postoperative complications and mortality [31]. Our findings also supported this theory. This might be because older persons are more susceptible to stresses, which can lead to the dysregulation of numerous systems and a decreased physiological reserve [32]. Numerous underlying conditions, including heart disease, hypertension, diabetes, and cerebrovascular disease, are common in elderly people and increase the risk of surgery.

Elevated CAR (HR 1.476, 95% CI: 1.209 ~ 1.801) and BNP (HR 1.001, 95% CI: 1.000 ~ 1.001) at admission were identified as independent predictors of increased 1-year mortality in elderly patients with hip fractures. With respect to hip fractures in elderly individuals, traumatic stress may lead to elevated blood C-reactive protein levels [33]. High preoperative CAR values are linked to increased early postoperative mortality in older patients with hip fractures, according to Cacciola et al. [34]. As the CAR integrates both inflammatory and nutritional dimensions, it serves as a valuable prognostic marker reflecting the patient’s underlying physiological reserve [35]. For patients presenting with elevated CAR, meticulous surgical planning to minimize procedural trauma, blood loss, and operative time may help attenuate surgical stress and potentially improve outcomes.

Similarly, elevated BNP levels indicate cardiovascular stress and subclinical myocardial injury—frequently exacerbated by systemic inflammation and hemodynamic challenges accompanying hip fracture [36,37]. Even mild elevations in BNP have been linked to adverse cardiac events and increased mortality, underscoring its utility as a sensitive prognostic indicator beyond traditional risk factors [38]. Close perioperative monitoring of BNP trends may aid in the early identification of patients at high risk for cardiovascular complications, allowing timely intervention to mitigate postoperative adverse events.

Conversely, higher hematocrit (HCT) levels were associated with reduced mortality (HR 0.922, 95% CI: 0.872 ~ 0.975). When diagnosing anaemia, haematocrit (HCT), which represents the proportion of red blood cells in the total blood volume, is essential [39]. Maintaining an appropriate HCT helps prevent anaemia-related complications and has an indirect protective effect on reducing the risk of death in geriatric patients with hip fractures. Furthermore, it enhances the overall recovery process by supporting adequate oxygen transport to tissues and organs, which is crucial for wound healing, immune function, and overall physiological stability [40]. Monitoring and maintaining adequate HCT levels should be an integral component of perioperative management in elderly hip fracture patients to avoid related complications and support recovery.

The predictive model integrating CAR and BNP levels developed in this study can provide individualized perioperative management strategies for hip fracture patients with comorbid chronic obstructive pulmonary disease. High-risk patients identified by the model should be referred to a multidisciplinary care pathway involving specialists from geriatrics, respiratory medicine, and cardiology. This proactive intervention strategy facilitates comprehensive comorbidity management, close monitoring of cardiopulmonary complications, and timely implementation of targeted interventions to reduce their high mortality risk. As we only performed internal validation without external validation in an independent cohort, future studies need to prospectively validate this model externally and evaluate whether this stratified multidisciplinary care approach can effectively improve survival rates and functional outcomes in this vulnerable population.

### Limitations

This study has several inherent limitations. First, since this was a retrospective analysis conducted at a single medical institution, our research results may not be fully applicable to other medical settings or groups with different clinical practices and patient characteristics. Second, although we adjusted for a series of known covariates related to mortality, we cannot rule out the influence of unmeasured or residual confounding factors (such as comprehensive frailty scores, detailed nutritional status, or chronic obstructive pulmonary disease severity on the basis of pulmonary function tests). We attempted to partially reflect overall health status by including comorbidities and ASA classification, but these factors cannot completely replace these factors. Third, our identification of chronic obstructive pulmonary disease relied on the established clinical diagnosis recorded in the medical records. We did not exclude patients due to the lack of pulmonary function reports, and we acknowledge that this is an inherent limitation of using routine medical data for research. Fourth, the cause of death was not adjudicated, especially in relation to COPD. Therefore, even in patients with chronic obstructive pulmonary disease (COPD), we cannot distinguish whether mortality is directly caused by complications related to COPD (such as respiratory failure) or is caused mainly by other factors. These limitations highlight the necessity of conducting prospective, multicenter studies in the future, which should adopt standardized data collection methods, including objective assessment of COPD severity and frailty, as well as detailed determination of the causes of death, to verify our findings and further clarify the involved mechanism pathways.

## Conclusions

On the basis of the findings of this study, chronic obstructive pulmonary disease (COPD) was identified as a significant independent predictor of mortality in elderly patients with hip fracture after adjustment for potential confounders. This highlights the considerable impact of COPD on postoperative survival in this vulnerable group. Furthermore, increased age, higher CAR and elevated BNP levels were also associated with greater one-year mortality, whereas a higher haematocrit (HCT) was linked to improved survival. Using these predictors, a nomogram was developed to estimate the probability of one-year all-cause mortality following hip fracture surgery. This tool may assist clinicians in the early identification of high-risk patients, allowing timely implementation of individualized treatment strategies. Close monitoring of these risk factors, together with advances in clinical assessment, could help improve outcomes and prolong survival in this patient population.

## Supporting information

S1 Table Baseline characteristics of individuals in the training set and validation set. (DOCX)

S1 Fig Associations of admission variables with 1-year mortality in elderly hip fracture patients. (A-D) Restricted cubic spline plots showing the relationships between 1-year mortality and (A) age, (B) hematocrit (HCT), (C) the C-reactive protein-to-albumin ratio (CAR), and (D) brain natriuretic peptide (BNP). The dotted line represents the reference line where the risk ratio is equal to 1. (E-H) Kaplan‒Meier survival curves after dividing patients into high- and low-level groups on the basis of the optimal cut-offs identified in panels A-D for (E) age, (F) HCT, (G) CAR, and (H) BNP.
(TIF)
