# Supplementary material for: Impact of chronic obstructive pulmonary disease on mortality in elderly patients with hip fracture: A retrospective cohort study
Source: PLoS One. 2026 Jan 6;21(1):e0340474. doi: 10.1371/journal.pone.0340474 (PMC12774376; doi:10.1371/journal.pone.0340474)
Supplement: S1 Table — (DOCX) [file pone.0340474.s001.docx]

**S1 Table. Baseline characteristic of individuals in training set and validation set.**

| Variables | Total  (n = 537) | Training set  (n = 376) | Validation set  (n = 161) | *P* |
| --- | --- | --- | --- | --- |
|  |  |  |  |  |
| Age, years | 79.93 ± 7.20 | 79.99 ± 7.12 | 79.79 ± 7.42 | 0.765 |
| Sex, n (%) |  |  |  | 0.372 |
| Female | 219 (40.78) | 158 (42.02) | 61 (37.89) |  |
| Male | 318 (59.22) | 218 (57.98) | 100 (62.11) |  |
| BMI, kg/m^2^ | 22.88 ± 3.80 | 22.78 ± 3.72 | 23.10 ± 3.98 | 0.379 |
| Type of fracture, n(%) |  |  |  | 0.713 |
| Femoral neck fracture | 267 (49.72) | 185 (49.20) | 82 (50.93) |  |
| Intertrochanteric fracture | 270 (50.28) | 191 (50.80) | 79 (49.07) |  |
| The time from injury to admission,h | 72.50 ± 134.60 | 74.10 ± 133.46 | 68.76 ± 137.55 | 0.674 |
| The time from admission to surgery,h | 125.43 ± 78.37 | 125.31 ± 77.40 | 125.71 ± 80.85 | 0.957 |
| COPD, n(%) |  |  |  | 0.780 |
| Yes | 149 (27.75) | 103 (27.39) | 46 (28.57) |  |
| No | 388 (72.25) | 273 (72.61) | 115 (71.43) |  |
| Hypertension, n (%) |  |  |  | 0.657 |
| Yes | 248 (46.18) | 176 (46.81) | 72 (44.72) |  |
| No | 289 (53.82) | 200 (53.19) | 89 (55.28) |  |
| Coronary artery disease, n (%) |  |  |  | 0.934 |
| Yes | 128 (23.84) | 90 (23.94) | 38 (23.60) |  |
| No | 409 (76.16) | 286 (76.06) | 123 (76.40) |  |
| Diabetes, n (%) |  |  |  | 0.734 |
| Yes | 115 (21.42) | 82 (21.81) | 33 (20.50) |  |
| No | 422 (78.58) | 294 (78.19) | 128 (79.50) |  |
| Cerebral infarction, n (%) |  |  |  | 0.768 |
| Yes | 295 (54.93) | 205 (54.52) | 90 (55.90) |  |
| No | 242 (45.07) | 71 (44.10) | 171 (45.48) |  |
| Pulmonary arterial hypertension, n (%) |  |  |  | 0.972 |
| Yes | 37 (6.89) | 11 (6.83) | 26 (6.91) |  |
| No | 500 (93.11) | 150 (93.17) | 350 (93.09) |  |
| Surgical type, n (%) |  |  |  | 0.737 |
| Joint replacement | 226 (42.09) | 160 (42.55) | 66 (40.99) |  |
| Internal fixation | 311 (57.91) | 216 (57.45) | 95 (59.01) |  |
| Method of anesthesia, n (%) |  |  |  | 0.422 |
| Intraspinal anesthesia | 170 (31.66) | 123 (32.71) | 47 (29.19) |  |
| General anesthesia | 367 (68.34) | 253 (67.29) | 114 (70.81) |  |
| ASA, n (%) |  |  |  | 0.528 |
| Ⅰ-Ⅱ | 269 (50.09) | 185 (49.20) | 84 (52.17) |  |
| Ⅲ-Ⅳ | 268 (49.91) | 191 (50.80) | 77 (47.83) |  |
| Duration of surgery,h | 97.31 ± 36.07 | 98.39 ± 36.32 | 94.81 ± 35.48 | 0.293 |
| Intraoperative fluid infusion volume, ml | 1385.69 ± 444.78 | 1376.73 ± 439.34 | 1406.61 ± 457.92 | 0.476 |
| HB, g/L | 112.81 ± 18.56 | 112.93 ± 18.40 | 112.53 ± 18.98 | 0.819 |
| WBC, 10^9^/L | 7.60 ± 2.56 | 7.59 ± 2.67 | 7.64 ± 2.27 | 0.835 |
| RBC, 10^12^/L | 5.12 ± 2.81 | 5.20 ± 2.88 | 4.94 ± 2.64 | 0.313 |
| HCT, % | 33.46 ± 5.57 | 33.50 ± 5.57 | 33.35 ± 5.57 | 0.764 |
| NLR | 6.84 ± 4.11 | 7.05 ± 4.32 | 6.36 ± 3.55 | 0.076 |
| CAR | 1.60 ± 1.18 | 1.63 ± 1.23 | 1.54 ± 1.06 | 0.454 |
| Sodium, mmol/L | 137.22 ± 4.08 | 137.14 ± 4.23 | 137.41 ± 3.69 | 0.473 |
| Potassium, mmol/L | 3.90 ± 0.50 | 3.92 ± 0.55 | 3.87 ± 0.35 | 0.250 |
| BNP, pg/mL | 242.80 ± 347.18 | 246.11 ± 378.19 | 235.09 ± 261.71 | 0.736 |
| Pneumonia, n(%) |  |  |  | 0.061 |
| Yes | 110 (20.48) | 69 (18.35) | 41 (25.47) |  |
| No | 427 (79.52) | 307 (81.65) | 120 (74.53) |  |
| Anemia, n(%) |  |  |  | 0.708 |
| Yes | 207 (38.55) | 143 (38.03) | 64 (39.75) |  |
| No | 330 (61.45) | 233 (61.97) | 97 (60.25) |  |
| Urinary system infection, n(%) |  |  |  | 0.911 |
| Yes | 39 (7.26) | 27 (7.18) | 12 (7.45) |  |
| No | 498 (92.74) | 349 (92.82) | 149 (92.55) |  |
| Respiratory failure, n(%) |  |  |  | 0.087 |
| Yes | 15 (2.79) | 14 (3.72) | 1 (0.62) |  |
| No | 522 (97.21) | 362 (96.28) | 160 (99.38) |  |
| Heart failure, n(%) |  |  |  | 0.135 |
| Yes | 147 (27.37) | 110 (29.26) | 37 (22.98) |  |
| No | 390 (72.63) | 266 (70.74) | 124 (77.02) |  |
| Acute Cerebral infarction, n(%) |  |  |  | 0.279 |
| Yes | 86 (16.01) | 56 (14.89) | 30 (18.63) |  |
| No | 451 (83.99) | 320 (85.11) | 131 (81.37) |  |
| Acute Myocardial Infarction, n(%) |  |  |  | 0.712 |
| Yes | 13 (2.42) | 8 (2.13) | 5 (3.11) |  |
| No | 524 (97.58) | 368 (97.87) | 156 (96.89) |  |
| Traumatic cholecystitis, n(%) |  |  |  | 0.556 |
| Yes | 9 (1.68) | 5 (1.33) | 4 (2.48) |  |
| No | 528 (98.32) | 371 (98.67) | 157 (97.52) |  |
| Delirium, n(%) |  |  |  | 0.851 |
| Yes | 35 (6.52) | 25 (6.65) | 10 (6.21) |  |
| No | 502 (93.48) | 351 (93.35) | 151 (93.79) |  |
| Atrial fibrillation, n(%) |  |  |  | 0.998 |
| Yes | 40 (7.45) | 28 (7.45) | 12 (7.45) |  |
| No | 497 (92.55) | 149 (92.55) | 348 (92.55) |  |
| DVT, n(%) |  |  |  | 0.390 |
| Yes | 147 (27.37) | 107 (28.46) | 40 (24.84) |  |
| No | 390 (72.63) | 269 (71.54) | 121 (75.16) |  |
| 1-year all-cause mortality,n(%) |  |  |  | 0.739 |
| Yes | 57 (10.61) | 41 (10.90) | 16 (9.94) |  |
| No | 480 (89.39) | 335 (89.10) | 145 (90.06) |  |
| Values are presented as mean±standard deviation,median and quartiles, or number (percentage) as appropriate, SD Standard deviation  ASA:American Society of Anesthesiologists Classification; HB: hemoglobin; WBC: white blood cell; RBC: red blood cell; HCT: hematocrit; BNP: Brain natriuretic peptide; NLR:Neutrophil/lymphocyte count ratio; CAR: CRP/albumin ratio;DVT: deep venous thrombosis | | | | |
